# The kidney tight junction (Review)

**DOI:** 10.3892/ijmm.2014.1955

**Published:** 2014-10-01

**Authors:** JIANGHUI HOU

**Affiliations:** Washington University Renal Division, St. Louis, MO 63110, USA

**Keywords:** tight junction, ion channel, scanning ion conductance microscopy, calcium signaling, kidney, epithelium

## Abstract

The tight junction is an important subcellular organelle which plays a vital role in epithelial barrier function. Claudin, as the integral membrane component of tight junctions, creates a paracellular transport pathway for various ions to be reabsorbed by the kidneys. This review summarizes advances in claudin structure, function and pathophysiology in kidney diseases. Different claudin species confer selective paracellular permeability to each of three major renal tubular segments: the proximal tubule, the thick ascending limb of Henle’s loop and the distal nephron. Defects in claudin function can cause a wide spectrum of kidney diseases, such as hypomagnesemia, hypercalciuria, kidney stones and hypertension. Studies using transgenic mouse models with claudin mutations have recapitulated several of these renal disease phenotypes and have elucidated the underlying biological mechanisms. Modern recording approaches based upon scanning ion conductance microscopy may resolve the biophysical nature of claudin transport function and provide novel insight into tight junction architecture.

## 1. Tight junction architecture

### A traditional view based upon electron microscopy

The tight junction in thin section electron microscopy is composed of a series of direct membrane contacts (1). Freeze-fracture electron microscopy reveals the membrane protein interactions at the tight junction as a branching and anastomosing reticulum of ‘fibrils’ or ‘strands’ on the protoplasmic fracture face (P face) (2). These fibrils have been demonstrated to be partly composed of integral membrane proteins directly involved in cell-cell interaction. Deep-etch microscopy and subcellular fractionation expose a matrix of densely packed globular proteins on the cytoplasmic surfaces of the junctional membranes that are detergent-resistant and sandwiched between the membrane surface and the actin cytoskeleton (3).

It is well known that the tight junction is a highly dynamic membrane structure whose strands are capable of substantial reorganization. Mechanical forces exerted upon the intestinal epithelium (4) or the mammary epithelium (5) cause the tight junction strand to elongate horizontally. The Sertoli cell tight junction exhibits even more dramatic dynamic behaviors during spermatogenesis. The spermatocyte migrates across the Sertoli cell tight junction through coordinated events of opening and re-establishing the tight junction strands (6).

### Molecular components

The known integral membrane proteins of the tight junction include occludin (7), junctional adhesion molecules (JAMs) (8) and claudins (9).

Occludin was the first tight junction integral membrane protein to be identified [Furuse *et al* (7)]. It has four transmembrane domains, two extracellular loops and long amino- and carboxy-terminal cytoplasmic domains. Occludin is ubiquitously expressed by virtually all epithelial cells and plays a number of physiological roles not confined to the tight junction. Occludin knockout (KO) mice have been shown to have no defect in tight junction function; however, they develop male infertility, chronic inflammation, hyperplasia of the gastric epithelium and calcification in the brain (10). Occludin may also play roles in cell polarity, directional migration and wound healing (11).

JAMs are a family of glycoproteins characterized by two immunoglobulin-like domains and are found in the tight junctions of both endothelial and epithelial cells, as well as on the membrane of circulating leukocytes and platelets (12). JAMs play diverse functional roles, regulating cell polarity, leukocyte/platelet-endothelium interactions and inflammatory responses (8).

**Figure 1:**
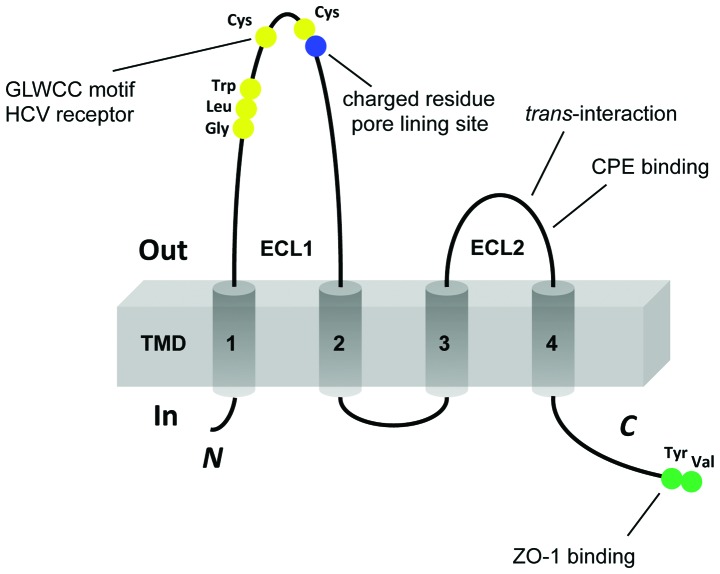
Claudin topology. The schematic cartoon illustrates the conserved regions in a claudin molecule. The first extracellular loop (ECL1) is important for paracellular ion selectivity and hepatitis C virus (HCV) binding; the second extracellular loop is important for claudin *trans*-interaction and *Clostridium perfringens* enterotoxin (CPE) binding. The YV motif in the carboxyl terminus is important for zonula occludens (ZO)-1 binding. TMD1–4, transmembrane domains 1 to 4. Modified from a previous study (14).

Claudins are tetraspan proteins consisting of a family of at least 26 members (13) ranging in molecular mass from 20–28 kDa. Claudins have four transmembrane domains, two extracellular loops, amino- and carboxy-terminal cytoplasmic domains and a short cytoplasmic turn (14) (Fig. 1). The first extracellular loop (ECL1) of claudin consists of ~50 amino acids with a common motif (-GLWCC; PROSITE ID: PS01346) (15), and intercalating negative (16,17) and positive (18,19) charges that contribute to paracellular ion selectivity. The GLWCC motif is critical as a receptor for hepatitis C viral entry (20). The charges in ECL1 regulate paracellular ion selectivity through electrostatic effects (21). The second extracellular loop (ECL2) consists of ~25 amino acids with a predicted helix-turn-helix motif that mediates *trans*-claudin interactions (22) and claudin interactions with the *Clostridium perfringens* enterotoxin (23). The carboxy-terminal domain of claudin contains a post-synaptic density 95/discs large/zonula occludens-1 (PDZ) binding domain (YV) that is critical for interactions with the submembrane scaffold protein, zonula occludens (ZO)-1 and correct localization in the tight junction (24).

### Crystal structure

**Figure 2:**
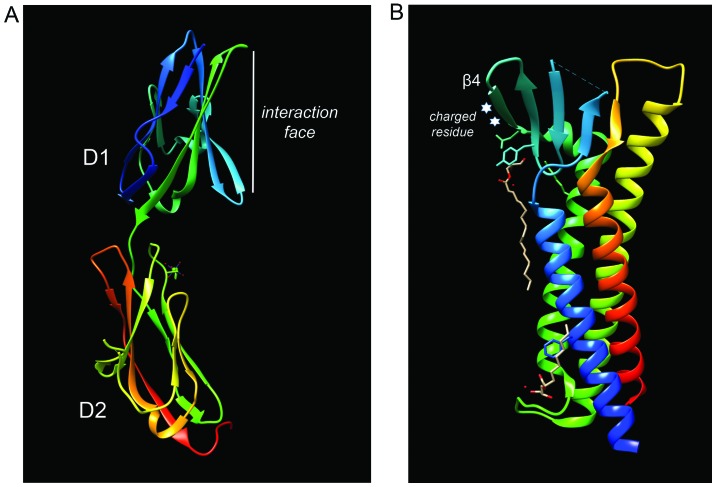
Junctional adhesion molecule (JAM) and claudin structures. 3D crystal structure of (A) monomeric JAM-1 ectodomain and (B) claudin-15 in ribbon representation. The color changes gradually from the N terminus (blue) to the C terminus (red).

The first resolved 3D crystal structure of a tight junction integral protein was that of JAM-1 (25). The ectodomain of JAM-1 protein consists of two immunoglobulin-like domains (D1 and D2) that are connected by a short conformationally restrained loop (Fig. 2A). Two JAM-1 molecules form a *cis*-dimer that is stabilized by a salt bridge and hydrophobic interaction between the D1 domains. Additional *trans*-interactions can be deduced from this crystal structure allowing the formation of a 2D network of JAM-1 molecules potentially important for tight junction architecture.

No crystal structure has been resolved for occludin. The first claudin crystal structure available was for claudin-15 (26), a claudin known to play roles in intestinal lumen formation (27). The transmembrane domain of claudin-15 forms a typical left-handed 4-helix bundle; the two extracellular loops contain prominent β-sheet structures (Fig. 2B). The fourth β-sheet of the ECL1 is enriched with charged residues that face the extracellular space and may play key roles in mediating paracellular ion permeation. A notable limitation of such a claudin monomer structure is that it provides little insight as to how claudins are polymerized to form the tight junction strand. Studies on a claudin related protein, IP39, from *Euglena gracilis* have hinted at how claudins are organized within the tight junction. In 2D crystals, IP39 forms a striated pattern of an antiparallel double-row network in which IP39 trimers are longitudinally polymerized (28).

## 2. Tight junction function

### A traditional view based upon flux assay

The simplest approach for recording tight junction permeability is the flux assay. As early as the 1970’s, small uncharged molecules, such as glycerol, dextran and inulin were used to measure the paracellular permeabilities and provide the first estimation of paracellular size selectivity (≤8 Å in diameter) (29,30). The use of an electrolyte as a tracer and the advances in electrophysiological recording technologies have allowed for more accurate and instantaneous measurements of tight junction permeabilities (17–19). Nevertheless, both the traditional flux assay and the modern electrophysiological approaches face a common obstacle, namely of how to differentiate the paracellular from the transcellular permeabilities. Various transcellular channel inhibitors can be used to reduce the transcellular permeability, but hardly eliminate it. Impedance measurements can isolate the paracellular permeability with mathematical deduction but are prone to recording errors as biological samples are not ideal electric circuitry (31,33). An even more serious concern over these primitive approaches is the lack of spatial resolution. In essence, the paracellular permeability is recorded over an area of several square centimeters that may contain several millions of channels. It will never be possible to resolve single channel permeability and dynamics using these approaches.

### Super-resolution recording

Due to the spatial orientation of the paracellular channels, traditional techniques, such as the patch clamp are not effective in achieving high-resolution recordings due to the leaky currents through cell-cell boundaries. The concept of ‘ion scanning’ is ideal for studying paracellular conductance based upon the following rationale: i) the approach is non-invasive as it employs a scanning electrode to measure local changes in current density or voltage amplitude; and ii) the approach can potentially differentiate paracellular from transcellular permeabilities as the electrode can be positioned above distinctive cellular features, such as the tight junction. Earlier studies have provided some preliminary measurements of the paracellular permeabilities using optical microscopy guided ion scanning (32–35).

The key limitation in optical microscopy-guided ion scanning is the lack of spatial resolution, both laterally to discern the subcellular structure, such as the tight junction and vertically to position the recording pipette over the biological sample. Scanning ion conductance microscopy (SICM) is a novel technology invented by Hansma *et al* (36) initially for material science. SICM utilizes a glass pipette similar in a patch clamp as a probe to image samples. By measuring the ion conductance between the glass pipette and the sample, Hansma *et al* (36) found that when the pipette is close to the sample, the ion conductance changes according to the pipette-sample distance. This phenomenon allows SICM to generate a topographical image of the biological sample surface. Such topographic images also allow SICM to position the pipette precisely over a subcellular biological feature.

**Figure 3:**
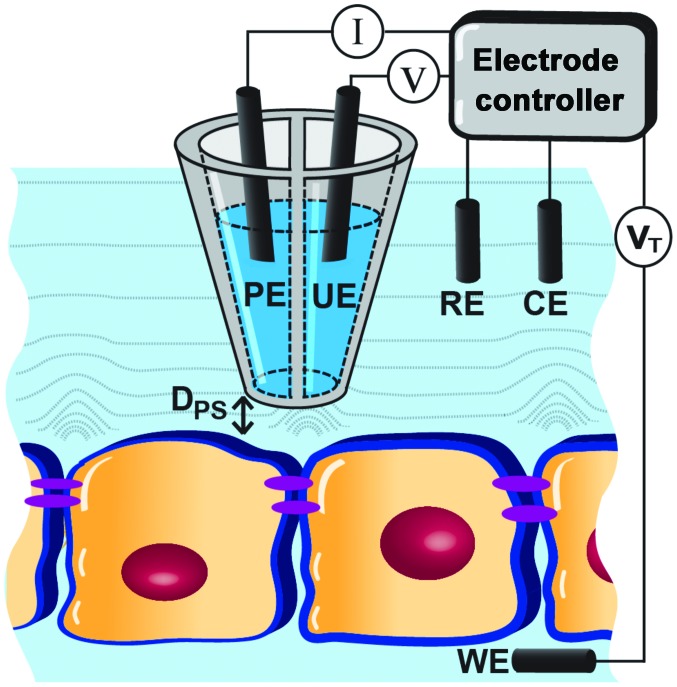
Schematic illustration of potentiometric-scanning ion conductance microscopy. A dual-barrel pipette is utilized to obtain topographic information of an epithelial monolayer and to measure local changes in transepithelial conductance over the cell-cell junction. PE, pipette electrode; UE, potential electrode; RE, reference electrode; CE, counter electrode; D_PS_, pipette-substrate distance; WE, working electrode. Modified from a previous study (66).

Although SICM was successful in non-contact imaging and pipette positioning, its application in membrane conductance measurement has been limited by the low signal-to-noise ratio for currant scanning. A breakthrough using SICM to record biological membrane conductance was made by Chen *et al* (37) in a modification to perform potentiometric-SICM (P-SICM). In P-SICM (Fig. 3), the circuit from pipette electrode (PE) to the reference electrode (RE) based upon monitoring the access current is used to draw a topographic image of the membrane surface and to position the pipette in a fixed distance above surface [maintaining constant pipette-to-surface distance (D_PS_)]. A second circuit from the potential electrode (UE) to the RE analyzes the potential variation in the vicinity of a membrane feature, such as a membrane channel or tight junction driven by transepithelial potential differences between the working electrode (WE) and the counter electrode (CE). A higher signal-to-noise ratio can be achieved in potential signals with the UE electrode than in current signals with the PE electrode (37). The ratio of potential change on UE and the current change on PE is in essence determined by the pipette resistance (38). This principle dictates that a smaller pipette with higher pipette resistance and smaller D_PS_ allows for a higher signal-to-noise ratio in P-SICM. Once a subcellular feature is located, a potential approach curve can be drawn against decreasing D_PS_ (38). The transmembrane conductance can be calculated according to the following equation, as previously described (37):

$$\text{G}=\frac{\text{E}}{\varrho {\text{V}}_{\text{T}}}=\frac{(\Delta {\text{V}}_{0.2\;\mu \text{m}}-\Delta {\text{V}}_{12.5\;\mu \text{m}})/\Delta \text{z}}{\varrho \Delta {\text{V}}_{\text{WE}}}$$

in which the electric field (E) is determined by dividing the potential difference (ΔV_0.2 μm_–ΔV_12.5 μm_) recorded at two distinct pipettte distances (D_PS_) by the vertical displacement of the pipette (Δz); ϱ is the specific resistance of the recording solution and V_T_ is the potential range applied at the WE to induce potential deflections (V_T_=100 mV, swept from −50 to +50 mV).

## 3. Tight junctions and renal physiology

### The glomerulus

The tight junction of glomerular parietal epithelial cells (PECs) is made of claudin-1, occludin and ZO-1 (39). In experimental crescentic glomerulonephritis, the gene expression level of claudin-1 is significantly reduced, accompanied by increases in paracellular permeability across the PEC barrier (39). The intercellular junction of glomerular visceral epithelial cells (podocyte) exhibits structural and functional transition during development. In the fetal glomerulus, the tight junction forms between immature podocytes; in the adult glomerulus, the tight junction gives way to a more specialized junction, known as the slit diaphragm. It is interesting to note that under various nephrotic conditions, the slit diaphragm retrogrades to the tight junction, coincident with the podocyte foot process effacement. The role played by claudin-1 in podocyte injury is best exemplified by diabetic nephropathy. In mice with streptozotocin-induced diabeties or obese (*db/db*) diabetic mice, claudin-1 gene expression has been shown to be profoundly upregulated in podocytes through SIRT1-mediated epigenetic mechanisms (40). The transgenic overexpression of claudin-1 in podocytes induces proteinuria in wild-type animals and aggravates streptozotocin-induced diabetic nephropathy (40).

### The proximal tubule

**Figure 4:**
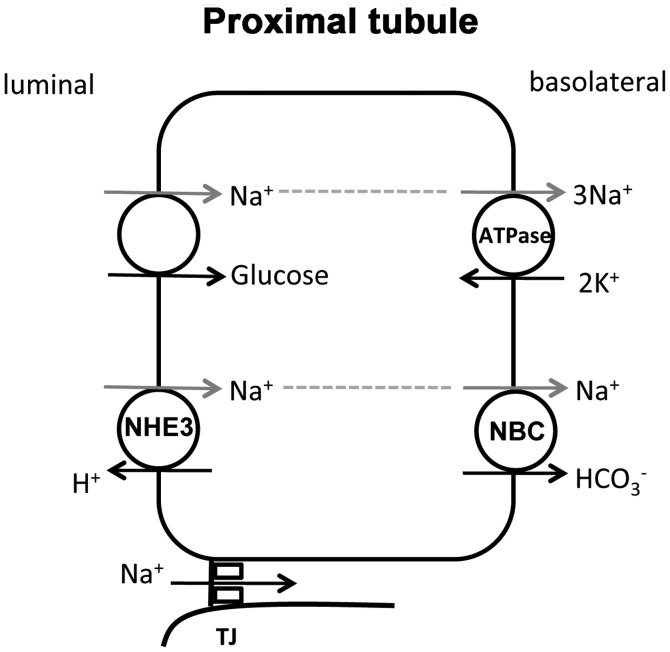
Ion transport pathways in the proximal tubule. Na^+^ is absorbed through the Na^+^/H^+^ exchanger (NHE3) and the Na^+^/glucose cotransporter localized in the luminal membrane and secreted into the basolateral side through the Na^+^/K^+^-ATPase and the Na^+^/HCO_3_^−^ contransporter (NBC). Additional Na^+^ can permeate through the tight junction (TJ) via the claudin-2 channels.

The major constituent of a tight junction in the proximal tubule is claudin-2. The genetic ablation of claudin-2 in mice has revealed transport defects specific to the proximal tubule, including decreases in the transepithelial reabsorption of Na^+^, Cl^−^ and water and increases in paracellular resistance (41). Under basal dietary conditions, claudin-2 KO mice present with hypercalciuria, but no renal loss of salt. As shown, these animals were however unable to cope with high salt infusion; their urinary excretion levels of both Na^+^ and Cl^−^ were significantly higher following infusion of 2% saline (41). The cellular mechanism responsible for paracellular salt transport in the proximal tubule is not completely clear. The transepithelial voltage is slightly negative with paracellular ion selectivity (P_Na_/P_Cl_) close to 1 (41). The majority of salt reabsorption therefore occurs through the transcellular pathway utilizing the Na^+^/H^+^-exchanger and the Na^+^/glucose-cotransporter (Fig. 4). Calcium is primarily handled by the paracellular pathway in this segment of the nephron and is purported to be regulated by flow rate (42). Water reabsorption in the proximal tubule is driven by the osmotic pressure differences following salt reabsorption and through the membrane water channel, aquaporin-1 (43). *In vitro* recordings have revealed claudin-2-dependent water transport in cultured renal epithelia, raising a tantalizing hypothesis that the tight junction is also permeable to water (44). Nevertheless, this hypothesis is somehow conflicting with the electric model of claudin-2 channel that permeates dehydrated ions (*vide infra*).

Claudin-2 is the most extensively studied claudin and the prototype for modeling the paracellular permeation pore. With the conventional Ussing chamber recording approach, Yu *et al* (21) have demonstrated the claudin-2 channel pore to be cation selective. The cation selectivity is mediated by the electrostatic interaction of dehydrated permeating cations with the negative charge on the side chain of Asp65 in claudin-2 protein. Chen *et al* (37) recorded the claudin-2 channel with the cutting-edge P-SICM technology and captured claudin-2 permeability within an area of nominal radii on the order of 265 nm. Both studies agree on the conductance density of claudin-2 channel at ~5–10 mS/cm^2^.

### The thick ascending limb of Henle’s loop (TALH)

The tight junction in the TALH comprises three important claudins: claudin-14, -16 and -19. They have all been connected to inherited calcium and magnesium imbalance diseases. Among them, claudin-16 was the first claudin gene identified from human renal disease with linkage analysis. Simon *et al* (45) discovered that mutations in claudin-16 cause a rare autosomal recessive disease, familial hypomagnesemia hypercalciuria and nephrocalcinosis (FHHNC; OMIM no. 248250). From patients with FHHNC with no claudin-16 mutation, Konrad *et al* (46) later found a second causal allele, the claudin-19 gene. FHHNC is characterized by severe renal loss of Ca^++^ and Mg^++^, growth retardation and kidney stones. Hou *et al* (47,48) created claudin-16 and -19 null mice and demonstrated that the loss of function in claudin-16 or -19 underlies the pathogenesis of FHHNC. It is interesting to note that claudin-16 and -19 proteins physically interact (49); the loss of one claudin causes the other to delocalize from the tight junction (48). Claudin-14 was identified from genome-wide association study as a major risk gene for hypercalciuric nephrolithiasis (kidney stone) (50). The claudin-14 protein was found to interact with claudin-16 and inhibit its transport function *in vitro* (51). The transgenic overexpression of claudin-14 in mice using a TALH-specific promoter has been shown to generate phenotypes resembling claudin-16 null animals, supporting the inhibitory role of claudin-14 in normal TALH function (52).

**Figure 5:**
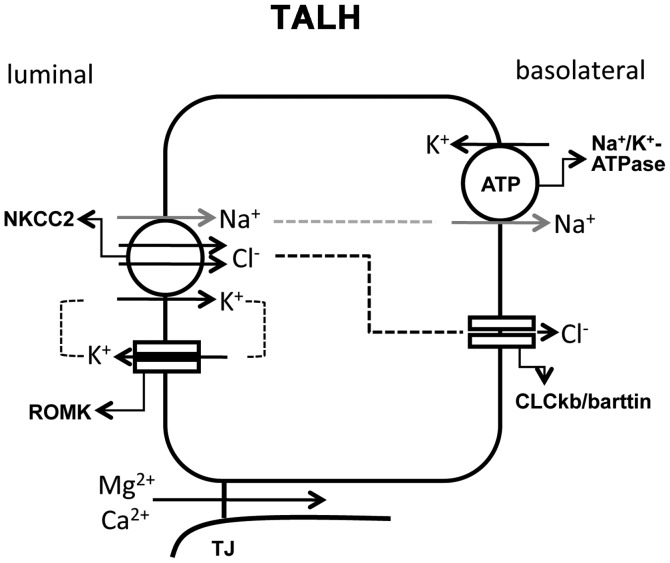
Ion transport pathways in the thick ascending limb of Henle’s loop (TALH). Na^+^, K^+^ and Cl^−^ are absorbed through the luminal membrane Na^+^/K^+^/2Cl^−^ cotransporter (NKCC2); Na+ is secreted into the basolateral side via the Na^+^/K^+^-ATPase; Cl^−^ is secreted into the basolateral side via the chloride channel Kb (ClCkb)/barttin channel; K^+^ is recycled into the luminal side through the renal outer medullary potassium channel (ROMK). Due to the continuous reabsorption of NaCl, a NaCl gradient develops from basolateral to luminal sides. This NaCl gradient passing through a cation selective tight junction (TJ) creates a lumen positive diffusion potential, which drives Mg^++^ and Ca^++^ reabsorption through the claudin-16 and -19 channels.

The tight junction in the TALH is highly permeable to cations and has a permeability ratio of Na^+^ to Cl^−^ (P_Na_/P_Cl_) of ~3–4 (53,54). Compatible with this concept, the transfection of claudin-16 into LLC-PK1 cells, whose tight junction expresses no endogenous claudin-16 and has low cation permeability, has been shown to profoundly increase the paracellular permeabilities to cations, including Li^+^, Na^+^, K^+^ and Mg^++^ (17). Mutations of claudin-16 found in patients with FHHNC have been shown to significantly reduce or completely abolish the cation selectivity (17). As shown in a previous study, when the TALH tubule was freshly isolated from mice and perfused *ex vivo*, the paracellular cation selectivity was lost in the claudin-16 null animals (47). As previously demostrated, transfection of claudin-19 into LLC-PK1 cells had no effect on cation permeability but significantly decreased the Cl^−^ permeability (49). The most telling finding was when claudin-19 and -16 were co-transfected, the tight junction became highly permeable to Na^+^ but not permeable to Cl^−^, generating a cation-selective pathway with P_Na_/P_Cl_ similar to that in native TALH epithelia (49). Due to the cation selective tight junction, the basolateral-to-luminal NaCl gradient will generate a lumen positive diffusion potential, which drives Ca^++^ and Mg^++^ reabsorption (Fig. 5). It is conceivable that when such paracellular cation selectivity is lost, Ca^++^ and Mg^++^ reabsorption is abolished.

Claudin-14 is a regulatory claudin that acts upon claudin-16 through physical interaction. As previously demonstrated, when co-transfected with claudin-16 into LLC-PK1 cells, the claudin-16-dependent P_Na_ was significantly reduced, while transfection with claudin-14 alone did not exert any significant effect on P_Na_ or P_Cl_ (51). The most important discovery of the physiological role of claudin-14 was made by Gong *et al* (51) and Gong and Hou (52) in extracellular calcium signaling. When animals were fed a high calcium diet, the renal gene expression level of claudin-14 was significantly upregulated; when the animals were fed a low calcium diet, claudin-14 gene expression was suppressed (51). Compatible with this result, claudin-14 null animals developed hypocalciuria and hypomagnesiuria under high dietary calcium conditions (51). Gong *et al* (51,55) and Gong and Hou (52) further revealed the signaling cascade to claudin-14. The extracellular calcium concentration is first sensed by the calcium sensing receptor (CaSR) expressed in the basolateral membrane of TALH cells (56); CaSR then regulates the transcription levels of two microRNA genes, miR-9 and miR-374 through nuclear factor of activated T-cells (NFAT) binding and histone deacetylation (52,55); the transcribed microRNA molecules recognize the partially complementary binding sites in the 3′-UTR region of the claudin-14 mRNA, suppress its protein translation and reduce its stability (51). Deriving from these discoveries, pharmacological intervention at various steps of CaSR signaling has proven to be an effective tool in regulating urinary Ca^++^ and Mg^++^ excretion levels (52,55).

### The aldosterone sensitive distal nephron (ASDN)

The ASDN in the kidney comprises the distal convoluted tubule (DCT), the connecting tubule (CNT) and the collecting duct (CD). While the tight junction in the DCT is very poorly understood, its role in the CNT and CD has been widely accepted as creating a paracellular pathway for Cl^−^ transport, known as the ‘chloride shunt’ (57). The chloride shunt is essential to electrical coupling with the epithelial sodium channel (ENaC) mediated Na^+^ reabsorption. The molecular component of chloride shunt has been identified by Hou *et al* (58) to include two claudin molecules, claudin-4 and -8. Using cultured CD cells, Hou *et al* (58) showed that the knockdown of claudin-4 or -8 significantly reduced the paracellular Cl^−^ permeability. Compatible with this concept, claudin-4 KO mice showed significant renal loss of Cl^−^ (59). More interestingly, claudin-4 and -8 physically interact; the knockdown of one claudin causes the other to delocalize from the tight junction (58). Claudin-7 is also found in the tight junction of CNT and CD. A constitutive KO model of claudin-7 showed severe renal loss of salt, volume and K^+^, accompanied by hyperaldosteronism (60). The loss of both cation and anion is consistent with the nonselective barrier role of claudin-7 in the CNT and CD. In cultured CD cells, Hou *et al* (58) found that the knockdown of claudin-7 resulted in a significant decrease in transepithelial resistance, but had no effect on ion selectivity.

**Figure 6:**
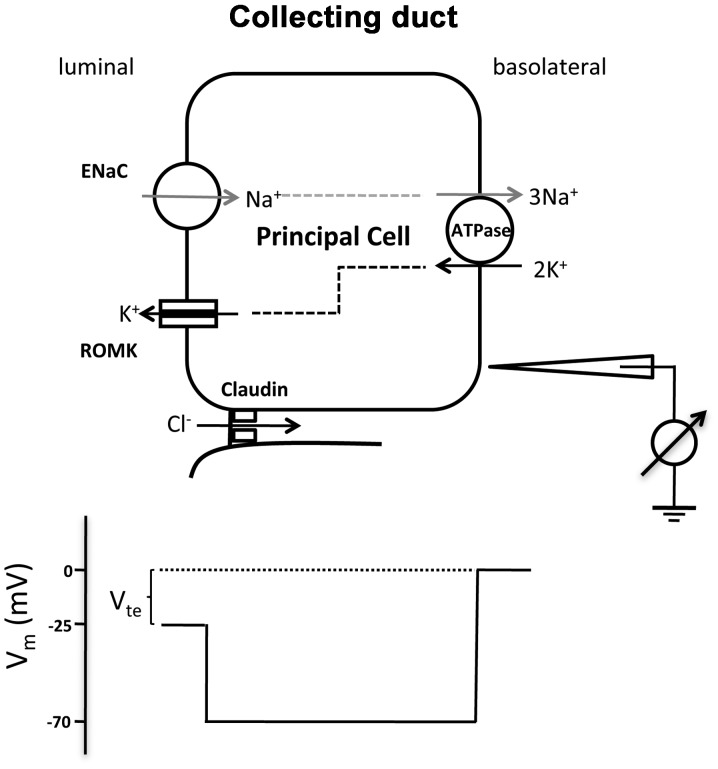
Ion transport pathways in the collecting duct. Na^+^ is absorbed through the epithelial sodium channel (ENaC); Na^+^ is secreted into the basolateral side via the Na^+^/K^+^-ATPase; K^+^ is secreted into the luminal side via the renal outer medullary potassium channel (ROMK). Because of the unilateral Na^+^ absorption, a lumen-negative potential develops, driving Cl^−^ absorption through the tight junction via claudin-4 and -8 channels. The membrane voltage (V_m_) trace depicts the virtual measurement by an electrode that is pushed from the basolateral side through the cell to the luminal side. In this example, the basolateral membrane voltage is −70 mV and the luminal membrane voltage is −45 mV, resulting in a transepithelial voltage (V_te_) of −25 mV with respect to the basolateral side.

The closure of the chloride shunt is expected to create a more negative transepithelial potential, depolarize the luminal membrane and inhibit ENaC mediated Na^+^ reabsorption (Fig. 6). Thus, the chloride shunt may in theory regulate the extracellular fluid volume and the blood pressure. In line with this hypothesis, WNK4, the mutation of which causes human hypertension in a rare inherited disorder, pseudohypoaldosteronism type II (OMIM no. 145260) (61), phosphorylates claudin-4 and increases chloride shunt permeability in cultured renal cells (62,63). The chloride shunt may be regulated by aldosterone. Le Moellic *et al* (64) demonstrated that aldosterone phosphorylated claudin-4 and increased paracellular Cl^−^ permeability in a rat CD cell line. In the distal colon, aldosterone upregulates claudin-8 transcriptional levels via the mineralocorticoid receptor (65). A similar mechanism may also exist in the ASDN.

## 4. Conclusions and future directions

### The mechanisms through which claudins oligomerize in the tight junction

Claudins function in pairs. In the TALH, claudin-16 and -19 interact and form the basic transport unit; in the CD, claudin-4 and -8 are both required for chloride shunt conductance. It is not yet known however, whether claudins adopt a dimeric structure in the tight junction. Resolving the oligomeric nature of claudins may provide key insight into the tight junction architecture.

### Recording single-channel conductance for paracellular channels

SICM represents a major breakthrough in paracellular channel recording. It can resolve paracellular conductance within submicron resolution. However, the claudin channel density in the tight junction is not easy to measure. Modern techniques, such as the super-resolution microscopy may provide a feasible tool.

### The mechanisms through which claudins are regulated in vivo

Phosphorylation and palmitoylation have both been reported as powerful means of regulating claudin trafficking and function. However, the mechanisms of claudin regulation have not yet been fully elucidated. The extracellular regulatory mechanism may be of particularly importance to the concept of a ‘druggable’ tight junction.

### The non-classic role of claudins

In addition to their classic role in the tight junction, claudins may play key roles in more specialized epithelial cells, such as podocytes. As several tight junction proteins have been found in the podocyte slit diaphragm and to interact with podocin, the biological role of tight junction proteins, including claudins in podocytes is particularly intriguing.

### The role of newly discovered claudins and claudin-related genes, such as tetraspanins

With novel genetic tools, such as transcription activator-like effector nucleases (TALENs) and clustered regularly interspaced short palindromic repeats (CRISPRs), it may become quicker and easier to generate mutant animal models for claudins and claudin-related genes. Such animal models may be of particularly importance to elucidating the mechanisms through which genetic mutations cause diseases.
